# Governor Clark and the Practice of Medicine

**Published:** 1895-04

**Authors:** 


GOVERNOR CLARK AND THE PRACTICE OF MEDICINE.—The high ground taken by Gov. Clark, of Arkansas, in support of an efficient medical act, his intelligent appreciation of the situation, and his support of the better element of the profession in their efforts to suppress quackery, is in such contrast with the ignorance, indifference or prejudice of most non-professionals as to deserve more than passing notice; we owe him thanks. All honor to him, we say.

The JOURNAL is indebted to Dr. J. A. Dibrell, of Little Rock, for a copy of the *Arkansas Gazette* containing Gov. Clark's message, vetoing a bill which the legislature had passed, repealing an efficient law requiring examinations by a State board; and recognizing diplomas, required examination only of non-graduates, and that too by county boards, of the judge's selection; restoring, in short, the old law which had been found by trial to be a mere farce. From the governor's message we select the following:

It is the opinion of myself, in common with a large part of the public, that the State board system has a tendency to make examinations more independent and impartial, and to make the granting of license depend more upon merit, by freeing such from the local influences of favoritism or prejudice. The matter to be investigated and notified to the public is the fact of the possession by the applicant of sufficient character and learning to take upon himself the responsibilities of a learned profession, which has to deal with the most vital interest of the human family, and not the question of whether or not he and his family connections are popular in the locality of his residence. I can not believe that it is the desire of any worthy applicant that this should be the case, but I prefer rather to believe that it is the desire of such as have not acquired a knowledge of the fundamental principles of the profession in a college of reputable standing, and to have the benefit before the public of an examition conducted by a board absolutely impartial and independent of personal or local considerations, and one whose membership is of such a character as to bear testimony that the successful applicant is qualified to the degree the public have a right to expect.

There was probably a time when a somewhat less stringent system of preliminary examination was tolerated in the interest of supplying remote localities with medical men even partially qualified. But this time has long since passed in Arkansas. The means of acquiring the necessary learning, supposing the native ability to exist, are now so plentifully to hand and at an expense fairly within the reach of any who choose to make the proper efforts, that it is discreditable to the pride of any candidate for the honors of the medical profession, to find him opposing any examination that a fair and intelligent board should subject him to.

That our State board is of such a character I am fully persuaded to believe. Because I believe the present law contributes to a greater degree than will the bill I herewith return, to elevate the standard of character and learning among the recruits to the ranks of the medical fraternity, and more largely confine the exercise of this important privilege to worthier persons at home, and will prevent an influx from other localities of a large number of persons who are prevented from imposing themselves upon the public there by laws similar to that which now stand upon our statute books, I am constrained to withhold my approval.

The legislature passed the act over the veto, nevertheless,—more's the pity.

